# Characterization of Isolated Extracts from *Justicia* Plant Leaves used as Remedy for Anemia

**DOI:** 10.3390/molecules25030534

**Published:** 2020-01-25

**Authors:** Jana Wood, Sayeda Yasmin-Karim, Michele Moreau, Rajiv Kumar, Janet Akwanwi, Atoh Derek, Fred Atoneche, John Kress, Wilfred Ngwa

**Affiliations:** 1 Brigham and Women’s Hospital, Dana-Farber Cancer Institute, Harvard Medical School, Boston, MA 02115, USA; Jana_Wood@dfci.harvard.edu (J.W.); syasmin-karim@bwh.harvard.edu (S.Y.-K.); michele_moreau@dfci.harvard.edu (M.M.); 2 Kennedy College of Sciences, University of Massachusetts Lowell, Lowell, MA 01854, USA; 3 College of Science, Northeastern University, Boston, MA 02115, USA; x4liebe@yahoo.com; 4 Sts Stephen and Paul’s Foundation, Bafut 2060, Cameroon; akwajanet@gmail.com; 5 PCC Health Services, Cameroon; abinwi21@gmail.com; 6 Department of Physics, University of Buea, Buea, Cameroon; fred.atoneche@ubuea.cm; 7 Department of Botany, MRC-166, National Museum of Natural History, Smithsonian, Institution, Washington, DC 20560, USA; tehandbifu@yahoo.com

**Keywords:** *Justicia* leaves, anemia, safety, nutraceutical, blood disorders

## Abstract

Indigenous populations use plants as an important healthcare resource or remedy for different diseases. Here, isolated extracts from *Justicia* (family Acanthanceae) plant leaves used in Africa as remedy for anemia are characterized by different methods to assess composition and potential nutritional or therapeutic value. Extracts from *Justicia* leaves were obtained by aqueous extraction, with further isolation by centrifuging and high-performance liquid chromatography. Extracts and isolated compounds were characterized by ultraviolet–visible (UV-Vis) spectroscopy and inductively coupled plasma mass spectrometry (ICP-MS). Hemoglobin activity was assessed using different hemoglobin assays (Cayman Chemical, and Sigma–Aldrich), as well as ELISA. In addition, the safety of the isolated samples was assessed in vitro and in vivo in mice. ICP-MS study results revealed many essential metabolites found in blood plasma. The UV-Vis spectroscopy results highlighted the presence of hemoglobin, with assays showing levels over 4 times higher than that of similar mass of lyophilized human hemoglobin. Meanwhile, in vivo studies showed faster recovery from anemia in mice administered with the isolated extracts compared to untreated mice. Moreover, in vitro and in vivo studies highlighted safety of the extracts. This study reveals the presence of high levels of elements essential for blood health in the isolated extracts from *Justicia* plant leaves. The findings inspire further research with the potential applications in food fortification, and as remedy for blood disorders like anemia, which disproportionally affects cancer patients, pregnant women, and populations in low- and middle-income countries.

## 1. Introduction

*Justicia* (family Acanthanceae) plants are consumed for different medicinal purposes, especially in pantropical regions of the world [1,2,3,4,5,6]. In many low- and middle-income countries (LMIC), local populations claim health benefits of consuming *Justicia* leaves to remedy anemia, albeit with little or no scientific evidence to support these claims [2,3,4]. More research characterizing extracts from these plants is needed to inform their use, with potential to develop evidence-based remedies for these populations, especially given the global prevalence of anemia and associated disparities.

In context, anemia affects over 2 billion people around the globe [7,8,9], with disparities where certain populations are disproportionately affected including 42% of pregnant women, and 47% of children younger than 5 years [7,10,11]. For patients with cancer, anemia occurs in about 30% at diagnosis [12], and increases significantly in severity during cancer treatment to up to 80% of cancer patients [13]. Many studies have reported on anemia as a prognostic factor of patient outcomes during cancer treatment like radiotherapy [12,14,15,16]. Cancer-related anemia also significantly impacts the quality of life of patients [17,18]. This negatively affects the ability of the patient to continue cancer treatment with consequential impact on treatment outcomes.

Current treatment options of anemia include blood transfusions, erythropoietic agents, anti-inflammatory therapies, iron supplementation, and nutritional interventions. Nutritional interventions include home fortification with micronutrient powders, fortification of staple foods and condiments, and activities to improve food security and dietary diversity [11,12,14]. Recent studies have also reported the highest anemia prevalence in Low- and Middle-Income Countries (LMIC) in Asia and Africa, where these conventional remedies are often not appropriate or accessible [9,10,11,19]. Meanwhile, with veganism on the rise, including in high income countries, there is a growing demand for innovative plant-based nutritional interventions and options for vegans and vegetarians who are more susceptible to anemia due to reduced consumption of meat products [20,21,22]. This highlights the need for new alternative remedies, including those with the potential to reduce global anemia disparities.

Modern medicine has benefited from studies of phytomedicines used by indigenous populations in developing remedies for disease with inadequate pharmacopeia. In this study, a survey was first conducted in the LMIC Cameroon, to establish use of *Justicia* leaves as a remedy for anemia. The components of extracts of *Justicia* leaves were then isolated and characterized to determine composition and activity elucidating such use. The results reveal high levels of metabolites essential for blood health and significant presence of hemoglobin, with little or no side effects. The findings provide impetus for further work developing potential applications in food fortification, and healthcare as a remedy for blood disorders like anemia and associated disparities.

## 2. Results

### 2.1. Establishing the Use of Justicia Leaves

An Institutional Review Board and Ethics Committee approved (IRB/005/11/2017) survey was conducted with informed consent of participants in the African country Cameroon, to establish the uses of *Justicia* and claimed healthcare benefits. The identity of the *Justicia* plants used was confirmed by a taxonomic specialist in the Department of Botany at the United States National Herbarium.

**Figure 1:**
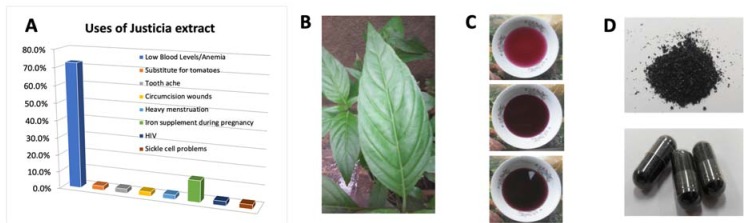
(**A**) Results from survey of plant extract use; (**B**) photo of *Justicia* leaves, (**C**) extract from leaves; (**D**) lyophilized powder from isolated leaves extract.

The survey results are shown in Figure 1A. The predominant use of *Justicia* leaves indicated by over 70% of participants was as a remedy for anemia. Other uses reported included in food preparations as a substitute for tomatoes in food preparation. Figure 1B shows sample *Justicia* plant leaf consumed in tea preparation (Figure 1C). A micronutrient powder developed from purified leaf extract is shown in Figure 1D. Altogether, the results established the use of extracts by populations in LMIC.

### 2.2. Characterization of Components of Leaf Extracts

Inductively coupled plasma mass spectrometry (ICP-MS) analysis of the leaf extracts revealed many essential components essential for human blood health [23]. Table 1 shows a list of metabolites with concentration levels. These include high levels of potassium (K), calcium (Ca), sodium (Na), magnesium (Mg), iron (Fe), etc. Also, using a blood gas analyzer, CO-oximeter, the electrolytes/metabolites concentrations for Na^+^, Ca^2+^, Cl^−^, and glucose were found to be 29 mmol/L (lower), 2.96 mmol/L (higher), 168 meq/L (higher), and 323 mg/dL (higher), respectively, in comparison to values of human arterial blood.

**Figure 2:**
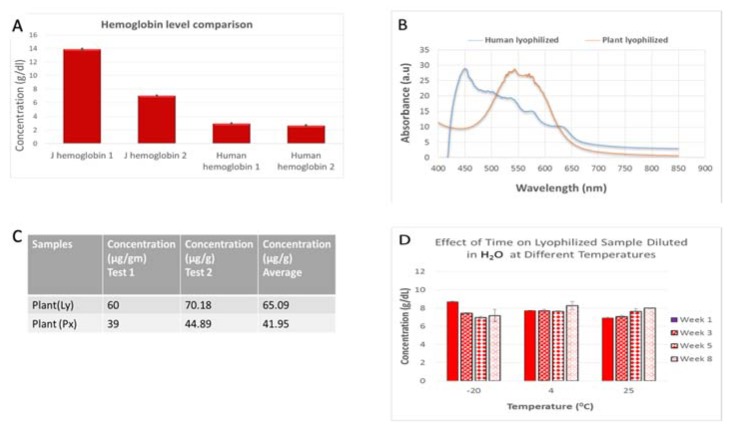
(**A**) Concentration of hemoglobin in isolated lyophilized powder from *Justicia* leaves reconstituted in water at different concentrations (J hemoglobin 1 and J hemoglobin 2) compared to concentration of hemoglobin in a similar mass of human hemoglobin lyophilized powder from MP Biomedicals (Human hemoglobin 1) and Sigma–Aldrich (Human hemoglobin 2). (**B**) Ultraviolet-visible (UV-VIS) spectroscopy of isolated *Justicia* plant powder compared to human hemoglobin lyophilized powder. (**C**) ELISA results further confirming hemoglobin activity in isolated lyophilized *Justicia* leaf extract dissolved in water at high concentration (Ly) and low concentration (Px); (**D**) Change in hemoglobin concentration levels as a function of different storage temperature over time.

Results obtained from different hemoglobin assay kits (Cayman Chemical, and Sigma–Aldrich) showed remarkably high hemoglobin concentrations in the isolated leaf extracts. For example, 30 mg of isolated and purified lyophilized extract reconstituted in 1 mL of sterile water showed 13.90 g/dl of hemoglobin compared to 2.97 g/dl for purified human hemoglobin lyophilized powder (MP Biomedicals) (Figure 2A), consistent with expectations for 30 mg of pure hemoglobin in one mL of water.

Meanwhile, the UV-VIS absorption spectra of the sample revealed results all consistent with previous findings for human hemoglobin. The absorption curve for human hemoglobin lyophilized powder (Sigma–Aldrich) showed a characteristic curve for methemoglobin [24], as would be expected (Figure 2B). Meanwhile, UV-VIS for the plant sample showed absorption peaks characteristic for hemoglobin (Figure 2B) [24]. The hemoglobin activity was further confirmed with ELISA (Figure 2C). The level of hemoglobin activity in isolated lyophilized powders remained stable over many weeks (Figure 2D) at different temperatures. The significance and context for these findings are discussed below.

### 2.3. Effect of Isolated Extracts on Cells In Vitro and In Vivo

**Figure 3:**
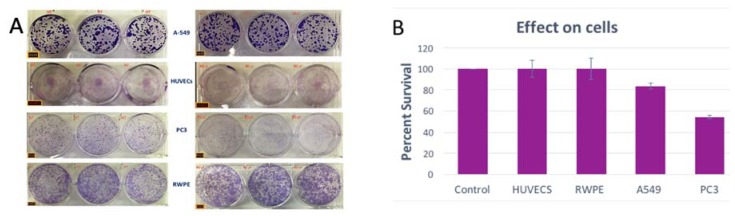
(**A**) Clonogenic survival assay results showing the effect of isolated plant extract on normal and cancer cells; (**B**) percent survival relative to controls. A549 = adenocarcinomic human alveolar basal epithelial cells; HUVECS = Human umbilical vein endothelial cells; PC3 = human prostate cancer cells; RWPE = normal prostate epithelial cell line.

To investigate the effects of the isolated purified extract on cells, investigations were carried out on different cancer cell lines, as well as normal cells to assess safety. The results (Figure 3A,B) showed little effect on normal cells: human umbilical vein endothelial cells, and RWPE prostate cell lines. Interestingly, the same amount of sample showed an inhibitory effect on PC-3 prostate cancer cells with clonogenic survival reducing to ca. 50%. More focused in vitro and in vivo mechanistic studies should elucidate such anti-cancer activity.

**Figure 4:**
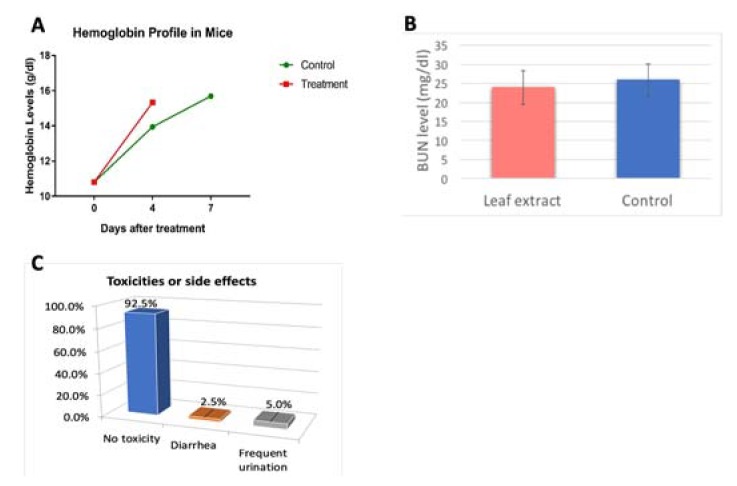
(**A**) Hemoglobin level comparison in mice treated with isolated plant leaf extracts versus control untreated mice; (**B**) Result of blood urea nitrogen (BUN) levels in animals administered with the isolated plant extract relative to that of animals in a control cohort. (**C**) Survey results on side effects by populations that consumed plant extract.

In addition, in vivo studies were carried out on female mice with chronic hemorrhagic anemia to assess any benefit of the isolated compound extract on anemia as highlighted in the survey (Figure 1A). Mice were divided into two cohorts. Cohort A was IP injected with the compound 100 mg/100g of body weight, and Cohort B (control) with saline. Blood was analyzed with ABL80 FLEX CO-OX (Radiometer) on days 2, 4 and 7 after the treatment to evaluate the hemoglobin and hematocrit levels. Results (Figure 4A) showed relatively more rapid increase in hemoglobin level in the group treated with the plant compound, supporting potential benefit as a remedy for anemia.

Meanwhile, no acute effect was observed of the isolated plant extract on kidney performance (Figure 4B. The results show comparable BUN levels for animals in mice administered with the isolated extract and mice in control cohort administered with deionized water which was used in sample solution. All mice maintained their body weights and appeared healthy prior to being euthanized. A separate cohort of mice also administered with sample was observed for over a month and remained as healthy as mice which were not treated. These observations are consistent with findings in survey of populations using the extracts, who reported minimal or no side effects (Figure 4C).

## 3. Discussion

The results in this study highlight high levels of vital blood components in *Justicia* plant extracts. The findings elucidate the potential benefits identified in a survey of people who report consuming extracts as a remedy for anemia. The high levels of hemoglobin may provide a possible explanation for anemia health benefits reported. One possible hypothesis that warrants investigation is that the benefit is from heme iron derived from the high level of plant hemoglobin, following hydrolysis in the stomach. Studies have established that heme iron has high bioavailability, is absorbed more efficiently than dietary inorganic iron, and is crucial for rapid generation of new red blood cells [25,26,27]. The heme iron in the extract could, therefore, be a potential nutritional remedy for anemia. Ongoing studies to establish a direct correlation between consumption of the leaf extracts and increase in hemoglobin levels in this population will be helpful in clearly establishing this benefit.

Meanwhile, the in vitro inhibitory effect observed on the growth of prostate cancer cell line (PC3) (Figure 3) deserves further investigations. A similar but not significant trend was observed for the lung cancer cell line (A549). Previous studies on *Justicia* plant extracts have highlighted anti-tumor activity [28,29]. More studies are needed here to further elucidate these observations and establish the mechanism of action, which could involve activity from other compounds present and contributing to the remedy effect.

Another question also worth further investigation is why these plants produce such high levels of hemoglobin. It is already well-established that plants, like humans, have hemoglobin genes, with hemoglobin genes widely expressed in eukaryotes [30,31,32,33,34,35,36]. In plants, symbiotic, non-symbiotic, and truncated hemoglobins have been reported with different roles, such as nitrogen-fixing symbiosis, facilitation of oxygen diffusion in rapidly respiring plant cells, or as oxygen sensor [31,34,36]. Despite gene expression in plants, the concentration level of hemoglobin in plant tissues (e.g., 0.03 g/dl) is orders of magnitude lower relative to human blood, which is typically above 10 g/dl [36]. The wide array of biotic and abiotic factors that may influence expression of hemoglobins in plants [37] makes it plausible that plants, under certain conditions, could adapt or develop high level of hemoglobin gene expression in its tissues. However, more studies are required to determine what factors may influence the development of such high hemoglobin concentrations in plants.

Besides hemoglobin, the high levels of elements like potassium, calcium, and magnesium (Table 1) proffer an abundant source of these mineral elements that could be leveraged for food fortification on as the source of micronutrients. This may be particularly beneficial for populations in LMIC where access to dietary supplements is limited especially, for elderly populations, as life expectancy increases.

Overall, the results elucidate the use of *Justicia* plant leaves, and justify more studies for further research with potential to develop evidence-based applications for treatment of blood disorders like anemia, which is particularly common in certain populations like pregnant women and kids [7,10,11]. Studies also show that anemia is common in patients undergoing cancer treatment like radiotherapy, chemotherapy, and surgery [12,14], and that this plays a role in the development of resistance to cancer treatment [38]. The results in this study justify further studies investigating the potential of formulations from this plant as a remedy for anemia observed in cancer patients which negatively affect therapy outcomes and quality of life [12,13]. Other populations that could benefit from these investigations are vegans or vegetarians [20,21,39,40] or populations in LMIC who have no access to enough meat diets from which heme iron is traditionally derived. Other potential applications that could develop include use in food coloring or nutraceuticals, and perhaps for developing blood substitutes from plants, which can mimic or fulfill some functions of human blood in trauma medicine.

## 4. Materials and Methods

### 4.1. Justicia Use Survey and Verification

An Institutional Review Board and Ethics Committee of the PCC Health Services approved (IRB/005/11/2017) survey was conducted in Cameroon with informed consent of participants. In this pilot survey 42 participants were asked about their use of *Justicia* extracts, the indications for which it was used, and about side effects they may have noticed after use. The survey was conducted from April 1, 2018 to July 31, 2018. Meanwhile, the identity of the *Justicia* plants used was confirmed by a taxonomic specialist in the Department of Botany at the United States National Herbarium. To authenticate the identity of the *Justicia* plant, lab protocols for generating DNA barcodes outlined by Kress et al. [41,42] and Kress and Erickson [43] were followed. A tissue sample, consisting of photosynthetic leaf material, of the plant under study was preserved by silica gel desiccation. A voucher specimen to document the plant from which the sample was taken was collected, dried, and deposited in the US National Herbarium (Washington, DC, USA).

### 4.2. Isolation and Analyses of Justicia Leaf Extracts

*Justicia* leaf sample was weighed and placed in known amount of purified water at 25 °C and rotated for 2 h to obtain extract. The extract was then centrifuged at 12,000 rpm, filtered and stored in vials. Lyophilization was carried out in a LYO-0.2 freeze drier (Tofflon Science and Technology). The following freeze drying protocol was used to lyophilize samples with a LYO-0.2 freeze drier (Tofflon Science and Technology): (1) freezing, −80 °C for 12 h with a cooling rate of 1 °C/min; (2) primary drying, −50 °C for 60 h at 3 Pa; and (3) secondary drying, 0 °C for 4 h and 20 °C for 8 h at 3 Pa. Further isolation and purification with HPLC was conducted with HPLC grade solvents. The lyophilized powder analysis was performed using an Agilent 1100 HPLC system with a diode array detector. A reverse phase ZORBAX Bonus-RP (2.1 mm × 150 mm, 2.7 µm) column was used for separation of the components of the extract. The analysis was performed using HPLC water (0.1% *v*/*v* Formic Acid) as mobile phase A and Acetonitrile (0.1% *v*/*v* Formic Acid) as mobile phase B. The HPLC analysis was performed at a column temperature of 250 °C. A 45 min gradient method was developed at a flow rate of 0.20 mL/min, with injection volume of 25 µl and the maximum absorbance wavelength of 575 nm. The gradient method consisted of 1% of mobile phase B maintained to 5 min. The gradient composition of mobile phase B was increased to 45% over 30 min and further increased to 75%. The mobile phase B was maintained at 75% for 5 min. Following the maintenance, the mobile phase B was dropped to 1% to the end of the run. In preparation of the stock, 10 mg of lyophilized powder was weighed and diluted with HPLC water to give a stock of 10 mg/mL concentration. The stock was sonicated and vortexed to give a clear dark purple solution. The solution was filtered through a 0.22 micron PVDF membrane filter. The stock was then diluted with HPLC diluents to give a 1 mg/mL sample concentration. The HPLC diluents was HPLC water: Acetonitrile (50:50, *v*:*v*).

Analysis with inductively coupled plasma mass spectrometry (ICP-MS) employed 0.5 mL of the samples filtered and mixed with 2% nitric matrix to make the total volume to 10 mL. Standards were made with Ca, Mg, Na, and K at a range of 10 down to 0.001 µg/mL. All other elements were listed at a range of 1.0 down to 0.0001 µg/mL. Blood gas analysis was performed with a state-of-the-art ABL80 FLEX CO-OX analyzer (Radiometer America).

Two separate human hemoglobin assay kits were used to assess the activity level of hemoglobin present in comparison to human hemoglobin as a control. First Cayman’s hemoglobin assay was performed following the instructions of the manufacturer (Cayman Chemical). Materials included hemoglobin sample buffer, hemoglobin detector, hemoglobin standard, 96-Well solid plate, and 96-Well cover sheet. Briefly, 20 μL of prepared samples was added to three wells of the 96-well plate for each sample. 180 μL of hemoglobin detector was added to each sample well. The plate was then covered with the plate cover and incubated in dark at room temperature for 15 min. The plate cover was then removed, and the absorbance read at 575 nm. The absorbance versus concentration curve generated using standard sample was then used to determine the concentration of hemoglobin in the sample compared to standard known samples and controls. Experiments were repeated for different dilutions. Other assays were performed per instructions of the manufacturer (Sigma–Aldrich), and lyophilized human concentrated hemoglobin (Sigma) was used as positive control along with Human Hemoglobin ELISA Kit from Abcam (ab157707).

Ultraviolet–visible spectroscopy was also performed, with spectra collected using an Agilent model 8453 UV-Vis scanning spectrophotometer over a wavelength range up to 1000 nm. The samples were measured against water as reference. All samples were used as prepared and loaded into a quartz cell for measurements.

### 4.3. In Vitro Studies

Primary human umbilical cord vein endothelium cells (HUVEC) were purchased from LONZA and were grown in a monolayer using Endothelial Cell Growth Medium with Bullet kit recommended by the company (LONZA). The cells were incubated at 37 °C with 5% CO_2_ under relative humidity. Trypsin from GIBCO were used to detach the cells and same amount of Trypsin Neutralizer (TN, GIBCO) was used to neutralize the Trypsin before adding the cell culture media. 

Adenocarcinomic human alveolar basal epithelial cells (A549), PC3 human prostate cancer cell lines, and RWPE-1 *Homo sapiens* normal prostate epithelial cell line were purchased from ATCC and cultured in RPMI-1640 media supplemented with 10% FBS. Cells were incubated at 37 °C in a humidified atmosphere (5% CO_2_, 95% air). 500 cells per well were seeded in 6-well plates on day one. Cells were treated with different concentrations of isolated extracts and experiments performed in triplicates.

Clonogenic cell survival assay was done following previously reported protocol [44]. Twenty four hours after treatment, all samples were reseeded as 10^4^ cells per well in 6 well plates in triplicates. Fourteen days after re-seeding colonies (>50 cells/colony) were fixed with 75% Ethanol in PBS and stained with 1% crystal violet (Sigma) and counted to calculate the survival.

### 4.4. Animal Studies

All studies were approved (approved protocol number 17-007) by the Dana-Farber Cancer Institute Institutional Animal Care and Use Committee and performed in accordance with National Institutes of Health guidelines. Chronic hemorrhagic anemia was induced in naïve healthy C57BL/6 female mice (Taconic) at ages between 10 to 12 weeks. To this end, small amounts of blood were drawn over a 2-week period, every other day three times per week via submandibular and Saphenous vein. Anemia levels were evaluated in blood every time after sampling until the mice reached determined anemia levels of hemoglobin (10 g/dl) or hematocrit (36 ± 1.0%). Mice were divided into two cohorts. Cohort A was IP injected with the isolated compound 100 mg/100 g of body weight, and Cohort B (control) with saline. Blood analysis was done with ABL80 FLEX CO-OX (Radiometer) on days 2, 4 and 7 days after the treatment to evaluate the hemoglobin and hematocrit levels. Mice were euthanized at each time point to collect bigger volumes of blood for further blood chemistry.

In studies on safety assessment, C57Bl6J mice (Jackson Laboratory, Bar Harbor, ME, USA) were utilized. Mice were divided into two cohorts. Cohort A mice were administered intraperitoneally with the compound at 180 mg/100 g body weight, as in previous studies assessing hemoglobin effect on kidney function [45]. Cohort B mice served as control with sterile water used. Mice were euthanized 24 h after the injections. Blood from euthanized mice was analyzed (at VRL-LLC lab) for blood urea nitrogen (BUN) levels to evaluate any acute effect on kidney function. Non-euthanized mice were observed over three weeks.

## Figures and Tables

**Table 1:** Essential mineral components of human blood found during ICP-MS analysis of isolated extract from Justicia leaves.

Analyte	Concentration (μg/mL)
Al	1.14
Ca	41.48
Cr	1.12
Fe	2.64
K	167.5
Mg	20.44
Mn	0.14
Na	63.44
P	3.04
Si	1.94
Sc	0.08
Ti	0.06
V	0.04
